# 24‐hour movement guidelines and weight status among preschool‐aged children in Bangladesh: A community‐level cross‐sectional study

**DOI:** 10.1002/brb3.3094

**Published:** 2023-05-29

**Authors:** Mohammad Sorowar Hossain, Enayetur Raheem, Anthony D Okely

**Affiliations:** ^1^ Department of Emerging and Neglected Diseases Biomedical Research Foundation Dhaka Bangladesh; ^2^ School of Environment and Life Sciences Independent University Dhaka Bangladesh; ^3^ Department of Digital Health Biomedical Research Foundation Dhaka Bangladesh; ^4^ School of Health and Society University of Wollongong Wollongong New South Wales Australia; ^5^ Illawarra Health and Medical Research Institute Wollongong Australia

**Keywords:** Childhood obesity, Bangladesh, Screen time, Sleep, Physical activity, 24‐hour movement activity

## Abstract

**Background:**

Increasing levels of urbanization and digitization in Bangladesh may be adversely associated with children's weight status and related movement behaviors. This study examined the prevalence of obesity, physical activity (PA), sedentary behavior, and sleep among young children from a district town in northern Bangladesh and identified factors associated with unhealthy weight status.

**Methods:**

Population‐based cross‐sectional study involving all kindergarten schools in Jamalpur District town. Schools and children aged 4–7 years were randomly selected and had their weight and height measured. Mothers completed a questionnaire on their child's PA, recreational screen time, and sleep and sociodemographic factors. Children's weight status was based on World Health Organization (WHO) categories. Meeting the PA recommendation was based on the WHO guidelines for children and adolescents, and meeting the sedentary behavior and sleep recommendations was based on the Canadian/Australian 24‐hour movement guidelines for children and young people.

**Results:**

A total of 585 children and their parents were included in the study. Overall, 15% of children were overweight or obese. Three‐quarters of children met the sleep guideline, and 50% met the PA guideline. Less than one third of children (31%) met the recreational screen time guideline, whereas 15% met all three guidelines. However, when adjusted for all predictors in the model, maternal education, family income, and child's age were significantly associated with overweight/obesity. Children with higher maternal education level were 2.3 times (AOR = 2.33, 95% CI: 1.19–4.78) more likely to be overweight/obese. Children in families with a higher monthly income had 1.9 times (AOR = 1.95, 95% CI: 1.14, 3.35) higher risk of being overweight/obese.

**Conclusions:**

Prioritizing maternal education (mother‐centric interventions) can help address the high levels of childhood obesity in Bangladesh.

## Graphical abstract

1. The proportion of children from an urban area in Bangladesh who were overweight or obese was 14%. 2. Less than one third (∼31 %) of children met the sedentary screen time guideline 3. Children with a higher level of maternal education and higher family income were more likely to be overweight/obese 4. Our findings can inform interventions and public health policies to reduce the increasing levels of child obesity in Bangladesh

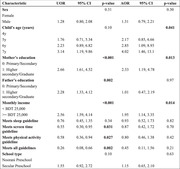

## 1 INTRODUCTION

The World Health Organization (WHO) considers childhood obesity as “one of the most serious public health challenges in the 21st century” (WHO, 2020a, 2020b). The prevalence of overweight and obesity in adolescents is defined according to the WHO growth reference for school‐aged children and adolescents. More than 41 million children under the age of 5 are estimated to be overweight worldwide. Over two thirds of these children live in low‐ and middle‐income countries (LMICs) (WHO, 2020a, 2020b), where the prevalence of childhood obesity has accelerated in recent years (Abarca‐Gómez et al., 2017).

Many LMICs, particularly in Southern Asia where there is a history of child malnutrition, now see childhood obesity as a major public health issue (Abarca‐Gómez et al., 2017; Hoque et al., 2014; Popkin et al., 2012). Childhood obesity is not well understood by parents in this region and is often perceived as a sign of good health (Hossain et al., 2019). Against the backdrop of managing undernutrition and life‐threatening communicable diseases, childhood obesity is often less prioritized in Southern Asia than undernutrition (Khan & Talukder, 2013).

Childhood obesity is influenced by many lifestyle, socioeconomic, genetic, and environmental factors (Gupta et al., 2012; Sahoo et al., 2015). Systematic reviews have shown that physical activity (PA) is negatively associated with adiposity in children (Carson et al., 2017; Jiménez‐Pavón et al., 2010; Poitras et al., 2016). More recently, time spent in 24‐hour movement behaviors (sleep, screen time, and PA) is associated with childhood obesity (Roman‐Viñas et al., 2016). WHO's Commission on Ending Childhood Obesity has also recognized the importance of movement behaviors in preventing obesity among children (WHO, 2016).

Bangladesh is a densely populated middle‐income country with a population of over 165 million. It is undergoing rapid unplanned urbanization whereby the proportion of people who live in urban areas will increase from 32% in 2020 to more than 50% by 2040 (Hayes & Jones, 2015). Concomitantly, the prevalence of childhood obesity (which includes overweight) has increased from 3.6% in 2000 to 9% in 2016 (Biswas et al., 2017; UNICEF, 2019). Data on the behavioral epidemiology of childhood obesity are scarce in Bangladesh (Biswas et al., 2017). Most studies among school children residing in the capital city, Dhaka, (population ≈ 18 M) have focused on the prevalence but not the risk factors or correlates of childhood obesity (Biswas et al., 2017). Overweight/obesity (OWOB) should be prevented as early as possible as children with excess weight are likely to become obese in adulthood (Geserick et al., 2018). Targeting preschool‐aged children could be highly effective as diet and physical habits are usually developed during preschool ages (Hodges, 2003). Identification of modifiable risk factors or correlates is critically important to develop intervention strategies for preventing childhood obesity in LMICs. This study aimed to: (a) determine the prevalence of OWOB among preschool children (4–7 years) in a district town and (b) to identify how OWOB is associated with sociodemographic characteristics and adherence to PA guidelines.

## 2 METHODS

### 2.1 Setting and participants

This cross‐sectional study followed the STROBE (strengthening the reporting of observational studies in epidemiology) statement for the transparent reporting of observational studies (von Elm et al., 2014). A list of kindergarten schools (*n* = 54) in the Jamalpur district town of Bangladesh (population 0.2 M, demographic survey 2011) was compiled and stratified into secular kindergartens and religious schools. Religious schools are referred to as *Noorani madrasas* where students from conservative Muslim families attend primarily for religious education. Notably, in Bangladesh, preschool‐level schools (KG‐I, KG‐II, privately run) are generally present in urban areas and are a part of primary schools. Government‐run schools start from the primary level (Standard‐I). In our study, two different types of schools (secular and religious backgrounds) were recruited from the district town where socioeconomically disadvantaged religious schools were located at the periphery of the urban areas. As such, the sample included children from urban and peri‐urban areas.

To be eligible, a school needed to be located within the Jamalpur district. Two special schools were excluded. Randomly stratified sampling was used to select eight secular and four religious schools. From each school, all children aged 4–7 years were invited to participate, and an information sheet and consent form were sent to their parents. If parents were interested in participating, they were asked to bring the consent form to the school and complete the parent assessments on campus. Ethical approval for this study was obtained from the Ethical Review Board of the Biomedical Research Foundation, Bangladesh (Memo no: BRF/ERB/2018/003). Informed written consent was taken from participating mothers before data collection. Data were collected between March 2018 and April 2018 by undergraduate and graduate students who were recruited from local universities and undertook training in how to administer the questionnaire and assess the child's weight and height.

### 2.2 Questionnaire

In this study, we conducted secondary analyses of a dataset from a previous study. The study used a questionnaire used was part of a larger survey that aimed to study maternal perception (Hossain et al., 2019) and correlates of childhood overweight/obesity in Bangladesh. A team of multidisciplinary experts, including public health researchers, statistician, and a representative of mothers, developed the questionnaire based on previously published literature and social context. The draft questionnaire was piloted and revised before field‐level data collection. Survey questions asked about the mother's age, education, occupation, marital status, husband's education and occupation, and monthly family income. Perception‐related questions (response categories: yes, no, or have not thought about it) included: Whether mothers felt stressed that their relatives might blame them if their child was thin. Mothers were asked to report the time their child spent (typically in a week) in sleep, PA, and screen time (TV, video, smartphone). We followed the WHO guidelines for physical activity for children and adolescents to determine the proportion who were adequately active. The Canadian and Australian 24‐hour Movement guidelines for Children and Youth were used to determine the proportion who met current guidelines for recreational sedentary screen time and sleep as there are no 24‐hour movement guidelines for children in Bangladesh (von Elm et al., 2014). The WHO guidelines recommend that children participate in an average of 60 min of moderate‐ to vigorous‐intensity physical activity (MVPA) per day. The Canadian and Australian guidelines recommend that, over a 24‐hour period, children spend less than 120 min in sedentary recreational screen time and obtain between 9 and 11 h of uninterrupted sleep per night. PA was operationalized as time spent in energetic play outside. For young children, the term energetic or active play has been used in recent PA guidelines developed for children in Australia and in the Asia‐Pacific (Loo et al., 2023; Loo et al., 2022; Okely et al., 2017). We have found that it is an easier concept for parents to understand than MVPA. Using energetic or active play to operationalize PA has been validated in a parent‐reported questionnaire against accelerometry and found to be adequate (Okely et al., 2009).

All data collectors underwent 2 days of training and orientation. This was provided by the same trainer using a set protocol. The trainer checked that data collectors administering the protocol in the same way and data collectors rehearsed the data collection procedures on one another and children as part of their training.

### 2.3 Outcome variables

Weight status: The height of the participating children was measured using a stadiometer (Seca, Hamburg, Germany) with a unit of 0.1 cm. Digital scales with a unit of 0.01 kg (Mega, Dhaka, Bangladesh) were used for assessing weight. Body mass index was calculated using height and weight measurements. Children removed their shoes and any heavy clothing before the height and weight measurements. All measures were taken once and then recorded.

### 2.4 Predictor variables

Predictor variables were PA, sedentary recreational screen time, sleep, and selected sociodemographic factors (child sex, child age, maternal and paternal education level, and monthly family income). Parents who wished to participate in the study attended the school and gathered in the school hall. All parents who attended were mothers and will be referred to as mothers from this point forward. A self‐ or interviewer‐administered questionnaire was completed by mothers in a large group (approximately 40–50 mothers). There was no mixing of mothers from secular and Noorani schools during data collection. Mothers were not allowed to discuss responses with each other. Mothers were asked to report the number of minutes per day, in a typical week, that their child spent in PA and sedentary recreational screen time and the time their child usually went to bed and woke up. PA was operationalized as energetic play outside and sedentary recreational screen time as any use of screen devices not for educational purposes or during school time. Mothers also reported their age, education level, occupation, marital status, husband's education and occupation, number of living children, and monthly family income. Mothers were also asked if they felt stressed that their relatives might blame them if their child was thin.

### 2.5 Statistical analyses

Children were classified as underweight, normal weight, overweight, and obese using the Centre for Disease Control and Prevention (CDC, Atlanta, GA, USA) standards (CDC, 2014). The WHO guideline for PA for children and adolescents was used to categorize children as active or inactive. This guideline recommends that children participate in at least an average of 60 min of MVPA per day (WHO, 2020a, 2020b). In the absence of global recommendations for time spent in sedentary behavior and sleep, we used the Australian and Canadian 24‐hour movement guidelines for children and young people to categorize children as meeting or not meeting the recommendation for sedentary recreational screen time and sleep. These guidelines recommend that, over a 24‐hour period, children spend less than 120 min in sedentary recreational screen time and obtain between 9 and 11 h of uninterrupted sleep per night (DOH, 2019; Tremblay et al., 2016).

Categorical variables were summarized as frequencies and percentages, whereas mean (SD) was used for continuous variables. Pearson's chi‐square test with continuity correction or Fisher's exact test was used as appropriate for categorical variables. The outcome variable was categorical with two levels—normal weight, overweight, or obese. Binary logistic regression models were used to examine the associations between the predictor and outcome variables. Unadjusted and adjusted odds ratios (adjusted for sex, parental education, monthly income, combined 24‐hour activities, peer pressure, and school type) were calculated. To report compliance with the Australian/Canadian 24‐hour movement guidelines, the proportions of children who met each of the three‐movement behavior guidelines (PA, sedentary screen time, and sleep) and who met all three guidelines were calculated.

## 3 RESULTS

### 3.1 Characteristics of study participants

A total of 649 children and their mothers from 12 preschools participated in the study, of which data from 585 participants (mother and child) were eligible for analysis after removing randomly missing data (9.9%). The mean age (SD) of included children and mothers was 5.5 years (SD 0.9) and 28.4 years (SD 4.6), respectively. Of the participants, 57.1% were boys. Nearly all mothers (98%) lived with their husbands and over 87% were housewives. Compared with children from Noorani preschools, children from secular preschools had a significantly higher socioeconomic status based on maternal education and family income (Table 1).

**TABLE 1:** Sociodemographic characteristics of parents.

**Characteristic**	**Overall**, *N* = 585	**Noorani preschool**, *N* = 188	**Secular preschool**, *N* = 397	***p*‐Value**
**Age of mother (mean ± SD)**	28.0 (25.0, 30.0)	28.0 (25.0, 30.0)	28.0 (25.0, 31.0)	.2
(Missing)	6	2	4	
**Mother's education**				**.031**
Primary	105 (18%)	39 (21%)	66 (17%)	
Secondary	174 (30%)	68 (36%)	106 (27%)	
Higher secondary	140 (24%)	40 (21%)	100 (26%)	
Graduate	160 (28%)	41 (22%)	119 (30%)	
(Missing)	6	0	6	
**Occupation**				.2
Housewife	506 (87%)	170 (90%)	336 (85%)	
Service	68 (12%)	16 (8.5%)	52 (13%)	
Entrepreneur	7 (1.2%)	2 (1.1%)	5 (1.3%)	
(Missing)	4	0	4	
**Marital status**				.4
Living with husband	567 (98%)	182 (97%)	385 (98%)	
Single/separated	14 (2.4%)	6 (3.2%)	8 (2.0%)	
(Missing)	4	0	4	
**Husband's education**				.11
Primary	70 (12%)	26 (14%)	44 (11%)	
Secondary	135 (24%)	53 (29%)	82 (21%)	
Higher secondary	148 (26%)	46 (25%)	102 (26%)	
Graduate	218 (38%)	60 (32%)	158 (41%)	
(Missing)	14	3	11	
**Husband's occupation**				.2
Service	306 (53%)	95 (51%)	211 (54%)	
Business	212 (37%)	65 (35%)	147 (38%)	
Farmer	15 (2.6%)	5 (2.7%)	10 (2.6%)	
Day labor	28 (4.9%)	13 (7.0%)	15 (3.9%)	
Other	13 (2.3%)	7 (3.8%)	6 (1.5%)	
(Missing)	11	3	8	
**Family income (monthly)**				**.016**
<10,000	205 (39%)	78 (46%)	127 (36%)	
10,000–24,999	153 (29%)	51 (30%)	102 (29%)	
25,000–49,999	162 (31%)	39 (23%)	123 (35%)	
≥50,000	0 (0%)	0 (0%)	0 (0%)	
(Missing)	65	20	45	
**Child's age (years)**				**<.001**
4	67 (11%)	16 (8.6%)	51 (13%)	
5	241 (41%)	84 (45%)	157 (40%)	
6	177 (30%)	70 (37%)	107 (27%)	
7	99 (17%)	17 (9.1%)	82 (21%)	
(Missing)	1	1	0	

*Note*: Mothers age = *t*‐test, marital status = Fisher's exact test.

### 3.2 Prevalence of underweight and overweight

Table 2 reports the prevalence of underweight, normal weight, overweight, and obesity by sex and type of school. Nearly one third of children were underweight, and 14% were overweight or obese. The prevalence of obesity was nearly twice as high among boys than girls (10% vs. 5.8%, *p* = .045). The proportion of children who were obese was twice as high among children from secular schools than their Noorani counterparts, (9.8% vs. 4.8%, *p* = .038). Overweight status differed across age groups (*p* = .033). Overweight prevalence was found to increase with children's age (6.2% at 5 years vs. 10% at 7 years) (Table 3).

**TABLE 2:** Weight status of children.

**Characteristic**	**Overall, N = 585**	**Girls**, *N* = 251	**Boys**, *N* = 334	***p*‐Value**	**Noorani preschool**, *N* = 188	**Secular preschool**, *N* = 397	***p*‐Value**
Overweight or obese	82 (14%)	31 (12%)	51 (15%)	.3	20 (11%)	62 (16%)	.11
Underweight	177 (30%)	70 (28%)	107 (32%)	.3	57 (30%)	120 (30%)	>.9
Normal	323 (55%)	149 (59%)	174 (52%)	.080	110 (59%)	213 (54%)	.3
Overweight	34 (5.8%)	17 (6.8%)	17 (5.1%)	.4	11 (5.9%)	23 (5.8%)	>.9
Obese	48 (8.2%)	14 (5.6%)	34 (10%)	.045	9 (4.8%)	39 (9.8%)	.038

**TABLE 3:** Children's weight status by age group.

**Characteristic**	**4 years**, *N* = 67	**5 years**, *N* = 241	**6 years**, *N* = 177	**7 years**, *N* = 99	***p*‐Value**
Overweight or obese	5 (7.5%)	30 (12%)	27 (15%)	20 (20%)	.10
Underweight	19 (28%)	74 (31%)	56 (32%)	28 (28%)	>.9
Normal	43 (64%)	136 (56%)	94 (53%)	50 (51%)	.3
Overweight	0 (0%)	15 (6.2%)	9 (5.1%)	10 (10%)	.033
Obese	5 (7.5%)	15 (6.2%)	18 (10%)	10 (10%)	.4

### 3.3 24‐hour movement behaviors

Table 4 reports the proportion of children who met the WHO PA guideline and the sedentary behavior, and sleep recommendations of the Canadian/Australian 24‐hour movement guidelines for Children and Youth. Three quarters of children (79%) met the sleep guideline, and half the children (50%) met the PA guideline. Less than one third of the children (31%) met the sedentary recreational screen time guideline. The differences between boys and girls in compliance with each of these guidelines were small and not statistically significant. Overall, only 15% of the children met all recommendations of the 24‐hour movement guidelines. The proportion of children from Noorani schools who met all three movement behavior recommendations was twice as high compared with that of secular schools (22 vs. 11, *p* = .001).

**TABLE 4:** Meeting 24‐hour movement guidelines.

Characteristic	Overall, *N* = 585	Girls, *N* = 251	Boys, *N* = 334	*p*‐Value	Noorani preschool *N* = 188	Secular preschool *N* = 397	*p*‐Value
Meets sleep guideline (9‐11 h/day)	458 (79%)	197 (80%)	261 (78%)	.5	146 (78%)	312 (80%)	.6
Meets screen time guideline (<2 h/day)	181 (31%)	85 (35%)	96 (29%)	.13	78 (41%)	103 (26%)	<.001
Meets physical activity guideline (Ave ≥1 h/day)	290 (50%)	123 (50%)	167 (50%)	>.9	103 (55%)	187 (48%)	.12
Meets all guidelines	86 (15%)	40 (16%)	46 (14%)	.5	42 (22%)	44 (11%)	<.001

### 3.4 Correlates of overweight/obesity

Table 5 reports the unadjusted and adjusted odds ratios between selected sociodemographic factors and meeting the PA, sedentary behavior, and sleep recommendations and overweight/obesity. Parental education (both mother and father), family income, child's age, meeting screen time and PA guidelines, and meeting all recommendations of the 24‐hour movement behavior guidelines were associated with overweight/obesity in the unadjusted models. However, when adjusted for all predictors in the model, maternal education, family income, and child's age were the only variables significantly associated with overweight/obesity. Children with higher maternal education were 2.3 times (AOR = 2.33, 95% CI: 1.19–4.78) more likely to be overweight/obese. Children in families with a higher monthly income were 1.9 times (AOR = 1.95, 95% CI: 1.14–3.35) more at risk of being overweight/obese. The odds of being overweight and obese were 2.9 times (AOR = 2.85, 95% CI: 1.09–893) higher for 6‐year‐old children and more than fourfold (AOR = 4.02, 95% CI: 1.46–13.1) higher for children aged 7 years compared to those aged 4 years.

**TABLE 5:** Unadjusted (UOR) and adjusted odds ratios (AOR) and 95% confidence intervals (95% CI) for sociodemographic and movement behaviors and overweight and obesity among children.

**Characteristic**	**UOR**	**95% CI**	***p*‐Value**	**AOR**	**95% CI**	***p*‐Value**
**Sex**			.31			.30
Female						
Male	1.28	0.80, 2.08		1.31	0.79, 2.21	
**Child's age (years)**			.10			**.041**
4						
5	1.76	0.71, 5.34		2.17	0.85, 6.66	
6	2.23	0.89, 6.82		2.85	1.09, 8.93	
7	3.14	1.19, 9.86		4.02	1.46, 13.1	
**Mother's education**			**<.001**			**.013**
0: Primary/secondary						
1: Higher secondary/graduate	2.66	1.61, 4.52		2.33	1.19, 4.78	
**Father's education**			**.002**			.97
0: Primary/secondary						
1: Higher secondary/graduate	2.28	1.33, 4.12		1.01	0.47, 2.19	
**Monthly income**			**<.001**			**.014**
<BDT 25,000						
≥BDT 25,000	2.56	1.59, 4.14		1.95	1.14, 3.35	
**Meets sleep guideline**	0.76	0.45, 1.35	.34	0.93	0.52, 1.73	.82
**Meets screen time guideline**	0.55	0.30, 0.95	**.031**	0.87	0.42, 1.72	.70
**Meets physical activity guideline**	0.58	0.36, 0.94	**.027**	0.80	0.46, 1.38	.42
**Meets all guidelines**	0.26	0.08, 0.66	**.002**	0.45	0.11, 1.56	.21
**School type**			.10			.63
Noorani preschool						
Secular preschool	1.55	0.92, 2.72		1.15	0.65, 2.10	

## 4 DISCUSSION

In this study, we found that there was a higher prevalence of obesity among boys and among children who attended secular preschools and higher maternal education and higher family income were associated with an increased likelihood of childhood overweight and obesity.

In our study, the combined prevalence of OWOB was 14%. A recent meta‐analysis has reported a similar prevalence (13%) for children aged 0–19 years in this country (Biswas et al., 2017). Nationally representative data on OWOB among school children are lacking in South Asian countries. Another meta‐analysis estimated that the combined prevalence of OWOB was 19.3% in India (Ranjani et al., 2016) and 14% in Pakistan (Warraich et al., 2009). A third meta‐analysis at the subcontinent level (India, Bangladesh, and Pakistan) found that the prevalence of OWOB was 2%–6% among rural children and adolescents, 16%–18% in average urban settings (nonaffluent), and 23%–36% among affluent urban children (Hoque et al., 2014). The rates of OWOB in wealthy countries are generally higher in rural areas and among people of lower socioeconomic status but the reverse is observed in LMICs (PHE,; Popkin et al., 2012). Higher rates of OWOB among urban affluent children residing in cities in South Asia are comparable to the situation in high‐income countries. These findings reinforce that major lifestyle changes are happening in rapidly urbanized South Asian countries, and OWOB is emerging as a major public health threat in these countries.

We found that the prevalence of obesity was nearly two times higher in boys than girls, whereas the prevalence of overweight was similar. These findings for obesity were similar to another study in Bangladesh (Das et al., 2013). Potential reasons for a higher proportion of obesity among boys include the patriarchal culture in Bangladesh where boys are generally more favored than girls. We also found that the prevalence of obesity was higher among children of secular schools compared to their Noorani counterparts. This disparity could be due to more socioeconomic disadvantaged conditions among families of Noorani school children whose parents have relatively less education and lower family income. This finding is consistent with another study in Bangladesh (Hossain et al., 2021).

In our study setting, the proportion of preschool‐aged children who met all PA, screen time, and sleep guidelines (15%) was similar to several other countries, including Australia (14.9%), China (15%), Sweden (19.4%), and Canada (12.7%) (Chaput et al., 2017; Cliff et al., 2017; Delisle Nyström et al., 2020; Guan et al., 2020). On the contrary, in our recent study conducted in Dhaka city (the most densely populated megacity on earth), a lower proportion (4.7%) of preschool‐aged children met all guidelines (Hossain et al., 2021). The proportion of children in the present study who met the screen time recommendation was found to be 31%, which was similar to Sweden (37.8%) and South Africa (48%) (Delisle Nyström et al., 2020; Draper et al., 2020). The proportion of children who met the PA guideline was approximately 50%, which was lower than several other countries, such as Sweden (∼90%), South Africa (84%), Australia (∼90%), China (65.4%), and Canada (61.8), indicating a substantial level of physical inactivity among children in a district town (Chaput et al., 2017; Cliff et al., 2017; Draper et al., 2020; Guan et al., 2020). More than half of the mothers who participated in our survey reported a lack of adequate neighborhood facilities such as playgrounds for their children. Our field‐level inspections revealed that there were no playgrounds in two thirds of the participating schools and none of the schools included sports activities in their curriculum. In this context, intervention strategies involving families and schools must be prioritized in developing countries like Bangladesh, which is undergoing rapid urbanization and digitization.

We found that a higher proportion of children from Noorani backgrounds met all guidelines as compared to that of secular schools. Cultural and socioeconomic factors might explain this finding. Noorani families are more economically disadvantaged and practice more conservative lifestyles. This means that their children would have less access to electronic media devices and spend more time playing outdoors

In the present study, higher maternal education and higher family income were associated with higher odds of being overweight/obese. These findings are consistent with our prior study in children under 5 years of age (Hossain et al., 2021), and studies reported from some LMICs (Chen et al., 2023; Gewa, 2010; Muthuri et al., 2016; Sahoo et al., 2015). The issue of higher maternal education regarding the risk of childhood obesity seems counterintuitive as higher maternal education is expected to positively influence children's diet and PA through various pathways, such as maternal knowledge and attitudes toward healthy eating, food availability and accessibility in the home, maternal modeling of eating behaviors, and participation in PA with children. Consequently, higher maternal education is associated with a reduced likelihood of adiposity among children in high‐income countries (White et al., 2022). On the other hand, maternal education at a higher level could potentially result in greater employment prospects and a higher income (Vollmer et al., 2017). However, this could also lead to a greater likelihood of having access to unhealthy food options, like fast food or convenience meals, and limited opportunities for PA due to time constraints (Muthuri et al., 2016). Additionally, cultural and societal attitudes toward obesity may contribute to its prevalence in some LMICs, where it is viewed as a desirable or healthy body type (Barlow, 2021). Urban areas with more affluent households tend to have greater availability of high‐calorie foods, which may further exacerbate the problem (Popkin et al., 2012).

However, interestingly, in our study setting, most participating mothers were stay‐home mothers (87%) who did not perceive childhood obesity as a health problem. Moreover, they were not knowledgeable about the risk factors and consequences of childhood obesity (Hossain et al., 2019). Therefore, prioritizing mother‐centric interventions is expected to be effective in preventing childhood obesity in LMICs, including Bangladesh (Hossain et al., 2020).

### 4.1 Limitations

Because of the stratified nature of sampling that was employed, the results are representative of the target population of 4–7‐year‐old school children in Jamalpur. The findings provide a baseline for similarly sized district towns in Bangladesh, a country with a large population with homogeneous characteristics with respect to language and sociocultural perspectives (MdB & Hossain, 2019). Due to the cross‐sectional nature of the study, it was not possible to make firm conclusions about correlates as the outcome (overweight and obesity) and exposure were assessed at the same time point and data on temporal sequence were not available. Adjusted odd ratios were derived using binary regression to incorporate the effects of confounders. However, this would not include the effect of any other factors not included in the study and in the analyses. For the assessment of PA and sleep, we relied on parent‐report, rather than device‐based measurements using accelerometry. Parent‐reports from high‐income countries have been shown to overreport time spent in PA and in sleep, although it is not clear if this is also true in LMICs. We operationalized MVPA as time spent in energetic play outside, which has been shown to be adequately valid (Okely et al., 2009), although it may have been difficult for mothers to report accurately about their child's daily play when their child was at school. We chose to use the guidelines for school‐aged children (rather preschool‐aged children) because only a very small number of children in the study were aged less than 5 years (*n* = 67). Moreover, our participating schools were mainly part of primary‐level schools where there were sections for preschool children (KG‐I, KG‐II).

## 5 CONCLUSIONS

This is the first study to examine associations between 24‐hour movement behaviors and childhood overweight/obesity among community‐level preschoolers in Bangladesh. In our study, children from affluent families and highly educated were more likely to be overweight/obese. Our findings provide a baseline for future studies and public health policies and interventions to reduce the increasing level of obesity in Bangladesh.

## CONFLICT OF INTEREST STATEMENT

The authors declare no conflict of interest.

## FUNDING INFORMATION

No external funding was available for this study.

### PEER REVIEW

The peer review history for this article is available at https://publons.com/publon/10.1002/brb3.3094.

## Data Availability

All data are available in the manuscript.
